# Preeclampsia: Narrative review for clinical use

**DOI:** 10.1016/j.heliyon.2023.e14187

**Published:** 2023-03-01

**Authors:** Paulino Vigil-De Gracia, Carlos Vargas, Joanne Sánchez, Jorge Collantes-Cubas

**Affiliations:** a Ginecología y Obstetricia, Hospital Complex of the Social Security Fund Hospital Doctor Arnulfo Arias Madrid (Complejo Hospitalario de la Caja de Seguro Social), Panama, Panama; b Hospital regional de Cajamarca, Cajamarca, Peru; c Hospital Complex of the Social Security Fund Hospital Doctor Arnulfo Arias Madrid (Complejo Hospitalario de la Caja de Seguro Social); Distinguished Researcher at the National System of Researchers, SENACYT, Panama, Panama

**Keywords:** Aspirin, High risk, Eclampsia, Preeclampsia, Prevention, Exercise and pregnancy

## Abstract

**Aim:**

Preeclampsia is a very complex multisystem disorder characterized by mild to severe hypertension.

**Methods:**

PubMed and the Cochrane Library were searched from January 1, 2002 to March 31, 2022, with the search terms “pre-eclampsia” and “hypertensive disorders in pregnancy”. We also look for guidelines from international societies and clinical specialty colleges and we focused on publications made after 2015.

**Results:**

The primary issue associated with this physiopathology is a reduction in utero-placental perfusion and ischemia. Preeclampsia has a multifactorial genesis, its focus in prevention consists of the identification of high and moderate-risk clinical factors. The clinical manifestations of preeclampsia vary from asymptomatic to fatal complications for both the fetus and the mother. In severe cases, the mother may present renal, neurological, hepatic, or vascular disease. The main prevention strategy is the use of aspirin at low doses, started from the beginning to the end of the second trimester and maintained until the end of pregnancy.

**Conclusion:**

Preeclampsia is a multisystem disorder; we do not know how to predict it accurately. Acetylsalicylic acid at low doses to prevent a low percentage, especially in patients with far from term preeclampsia. There is evidence that exercising for at least 140 min per week reduces gestational hypertension and preeclampsia. Currently, the safest approach is the termination of pregnancy. It is necessary to improve the prediction and prevention of preeclampsia, in addition, better research is needed in the long-term postpartum follow-up.

## 1 Introduction

Preeclampsia is a multisystemic disorder characterized by mild to severe hypertension during the second half of pregnancy or postpartum, leading to adverse pregnancy outcomes. It is estimated that approximately 76,000 women and half a million fetuses and neonates die each year from this disease worldwide [1]. The exact process that leads to the development of this pathology is unknown, and we can agree on the existence of three stages: the first is an anomalous invasion of the trophoblast into the spiral arteries, which generates an inadequate remodeling of the spiral arteries; the second stage then occurs, which is an alteration in the production of angiogenic and antiangiogenic factors. The time of onset is unknown, nor is it known with which alteration of these factors or at what gestational age this second stage begins and ends. Then, the third stage follows the clinical manifestation, which is very typical and widely known in this pathology [[2], [3], [4]]. Clinical manifestations can range from asymptomatic conditions to seizures and death [4]. Preeclampsia can be subdivided into early onset or late onset using 34 weeks as a cutoff or preterm or term using 37 weeks of gestation as a cutoff [1]. Approximately 80–90% are late onset or term preeclampsia and, in addition, of better evolution with fewer complications.

Despite much research aimed at predicting preeclampsia, it is not yet known with good or adequate precision how to predict this pathology, and we continue to rely on clinical risk factors [5]. In the last three decades, there has been much research on the prevention of preeclampsia, and we will analyze the main research and recommendations of specialized organizations. Acetylsalicylic acid (aspirin) at low doses remains the only drug measure with a moderate effect in preventing preeclampsia according to clinical risk factors [1,6,7].

The management of preeclampsia remains focused on a timely diagnosis and termination of pregnancy, of course that perinatal results are influenced by gestational age [4,5]. The use of antihypertensives is still elementary, especially in severe hypertension and the use of magnesium sulfate to prevent seizures [4,5,8]. Complications with preeclampsia vary greatly according to the study, ranging from no maternal or fetal complications to death of the mother and fetus or neonate [4,9].

The objective of this review is to update on preeclampsia, including its pathophysiology, diagnosis, clinical manifestations, prediction, prevention, maternal and perinatal complications, management, future scenarios and conclusions.

## 2 Search strategy and article selection criteria

PubMed and the Cochrane Library were searched from January 1, 2002 to March 31, 2022, with the search terms “pre-eclampsia” and “hypertensive disorders in pregnancy”. We cross-referenced these terms with the following: “pathophysiology”, “definition”, “prediction”, “prevention”, “management”, “clinical trials”, “aspirin”. We also look for guidelines from international societies and clinical specialty colleges and we focused on publications made after 2015.

## 3 Definition and classification of preeclampsia

Preeclampsia is a very complex multisystemic hypertensive syndrome typical of pregnancy. Over the years, the definition of this pathology has changed. Formerly, it was defined as the appearance of hypertension accompanied by proteinuria after 20 weeks of gestation. Today, the International Society for the Study of Hypertension in Pregnancy (ISSHP) proposes a broader definition, which is the most accepted internationally [1,10]. Society defines preeclampsia as the presence of systolic blood pressure greater than or equal to 140 mmHg or diastolic blood pressure greater than or equal to 90 mmHg in a pregnant woman at 20 or more weeks of gestation, with normal blood pressures before pregnancy. It must be verified in a minimum of 4 h later. In addition to hypertension, one or more of the following manifestations must have appeared recently [10]: Proteinuria: greater than or equal to 300 mg of proteinuria in 24-h urine sample or protein/creatinine ratio in a random urine sample greater than or equal to 0.3 mg/mg, or urine dipstick result of 2 or more for protein. Organ dysfunction: Acute kidney injury: creatinine greater than or equal to 1 mg/dL. Hepatic compromise: transaminases greater than 40 IU/L with or without pain in the right upper quadrant or epigastric pain. Neurological complications: persistent scotoma, severe headache, blindness, altered mental status, eclampsia, or clonus. Hematological disorders: platelet count lower than 150,000/μL, disseminated intravascular coagulation or hemolysis. Uterus-placental dysfunction: fetal growth restriction, altered umbilical artery Doppler or death.

There are several classifications of preeclampsia in clinical practice. According to the time of onset, it is divided into early and late, using 34 weeks of pregnancy as a cutoff point [1,11]. On the other hand, it is based on the presence or absence of signs and symptoms of severity. The American College of Gynecologists and Obstetricians (ACOG) [5] considers a diagnosis of preeclampsia with severity data as the presence of any of the following: Systolic blood pressure greater than or equal to 160 mmHg or a diastolic blood pressure greater than or equal to 110 mmHg, verified on two occasions at least 4 h apart, unless antihypertensive therapy is initiated before this; platelet count less than 100 × 109/L; elevation of liver enzymes to twice the upper limit of normal concentration, severe and persistent pain in the right upper quadrant or epigastric pain that does not resolve with medications; renal insufficiency with a serum creatinine concentration greater than or equal to 1.1 mg/dL or double the serum creatinine concentration in the absence of kidney disease; pulmonary edema; new onset headache that does not respond to medications; visual disturbances.

It should be noted that pregnant women with a previous diagnosis of hypertension with onset of proteinuria or maternal organ dysfunction after 20 weeks are classified as chronic hypertensive with added preeclampsia [4].

## 4 Pathophysiology of preeclampsia

When addressing the pathophysiology of preeclampsia, one must start from the fact that it is not completely known and that everything indicates a multifactorial cause, which is why it is known as the disease of “theories” because it is an enigmatic and elusive disorder.

It is known that the development of preeclampsia has maternal genetic, immunological, and inflammatory factors, leading to failure of placentation and intolerance between maternal, paternal (placental) and fetal tissues. These two major causes lead us to the focus of the pathophysiology, which is the reduction of utero-placental perfusion and therefore to utero-placental tissue ischemia, which in the end will result in generalized endothelial damage.

The theories that support this concept can be evidenced in the model by Odgen et al. [12], in which the clamping of the aorta below the renal arteries in nonpregnant dogs did not cause hypertension; in contrast, performing the same procedure in pregnant animals caused hypertension. Finally, this hypertension disappeared when performing hysterectomy, a concept that has been supported in other studies when clamping the uterine arteries in pregnant dogs, obtaining the same results [13].

This model of uteroplacental ischemia is supported by in vivo studies, numerous studies with injection of radioactive sodium and other radioactive tracers, in which it is observed that pregnant women without preeclampsia presented a utero-placental flow in term pregnancies of approximately 600 mL/min, but not in pregnant women with preeclampsia, where the flow decreased significantly and could fall up to 50% if it was severe preeclampsia, compared to mild preeclampsia [14,15]. Similar to a model in nonhuman primates, baboons, when performing uterine artery ligation, we observed findings similar to preeclampsia, such as hypertension, proteinuria, increased circulating concentrations of soluble vascular endothelial growth factor receptor 1 (sVEGFR-1; also known as soluble fms-like tyrosine kinase-1 [sFlt-1]) and endoglin. In addition, there is an increase in the concentrations of proinflammatory cytokines, such as tumor necrosis factor α (TNF-α) and interleukin (IL)-6 [16]. Another notable finding is that the administration of short interfering RNAs, which silence three of the sFlt-1 messenger RNA (mRNA) isoforms, was observed to suppress the overexpression of sFlt-1, consequently resulting in a reduction of hypertension and proteinuria. This leads to the hypothesis that the “toxin” that can be seen as responsible for hypertension may be sFlT-1 [13,17].

To date, it is impressive that the etiology of preeclampsia has defects in the placenta that lead to ischemia as its main component. However, the dysfunctional maternal cardiovascular system has recently been implicated as a significant factor in preeclampsia [11]. Both pathologies share common symptoms, hypertension, cerebral edema, and cardiac dysfunction.

The lack of physiological transformation of the spiral arteries, in which they maintain smooth muscle and narrow diameter, is believed to make these vessels prone to the effect of circulating vasoconstrictor agents; additionally, these vessels are more likely to develop atherosclerosis, causing the vessel lumen to be narrower, and therefore, making placental perfusion more compromised [18].

We can summarize that there is a link between preeclampsia and placental ischemia, as we have observed in studies in animal models [12], leading to hypertension and proteinuria; there is lower placental flow in pregnant women with preeclampsia [14]; failure of the physiological transformation of the spiral arteries; and an increase in the relationship between maternal placental growth factor (PlGF) and sFlt-1 [17,18].

## 5 Risk factors of preeclampsia

It is well known that preeclampsia has a multifactorial genesis. High-level, well-developed studies seem to converge on certain risk factors, behaving as a common denominator.

However, there are additional factors and complications that predispose women to have subsequent pregnancies with an increased probability of developing preeclampsia, such as spontaneous premature birth (1.1 to 1.8% if it is > 32 weeks and 3.2% if it is earlier than 28 weeks of gestation) and fetal growth of 2–3 standard deviations below the mean [19].

Previously, infectious diseases were associated with a risk factor for preeclampsia; however, they could not be confirmed. Currently, there are studies that indicate an association of preeclampsia and urinary tract infection [20] and certain specific reports in which it has been associated with malaria [21,22], cytomegalovirus [23], human immunodeficiency virus [24] and, more recently, infection by SARS-CoV-2 [14,25].

There are multiple studies on SARS-CoV-2 infection, being a recent pathology and a global problem, studies which we can cover through a meta-analysis, through which the association of SARS-CoV-2 and pregnancy with a significant increase in preeclampsia (OR, 1.58; 95% CI, 1.39–1.8), preeclampsia with severe characteristics (OR, 1.76; 95% CI, 1.18–2.63), eclampsia (OR, 1.97; 95% CI, 1.01–3.84) and HELLP syndrome (OR, 2.01; 95% CI, 1.48–2.97) was confirmed [25].

Diabetes mellitus has also been associated with preeclampsia in multiple studies, from systematic reviews (aRR, 3.56; 95% CI, 2.54–4.99) [26] to retrospective studies of 647,392 pregnancies (aOR, 1.29; 95% CI, 1.19–1.41) [27]. These findings have been supported by comparing them with studies showing a decreased risk of preeclampsia when treated with diet, metformin and insulin, including two systematic reviews [28,29].

Maternal age is an important risk factor. It is suggested that the risk of preeclampsia increases up to 30% for each year after 34 years of age. In a systematic review, it was established that maternal age ≥35: RR 1.2, 95% CI 1.1–1.3; maternal age ≥40: RR 1.5, 95% CI 1.2–2.0 [30]. It should be considered that older patients may present other additional risk factors, such as hypertension, diabetes mellitus, metabolic syndrome, and cardiac disease, which can induce the development of preeclampsia [30].

Body mass index with BMI> 25 kg/m^2^ [RR, 2.1, 95% CI 2.0–2.2], considered as overweight, and a BMI> 30 kg/m^2^ [RR, 2.8, 95% CI 2.6–3.1), considered as a range of obesity, act as a risk factor. By increasing body weight from 5 to 7 kg/m^2^, the risk of developing preeclampsia doubles [31]. We can corroborate this in a meta-analysis of 29 retrospective studies, with 1,980,761 participants and 67,075 cases of preeclampsia, where it was shown that pregnant women with a BMI> 30 kg/m^2^ (aOR, 2.93; 95% CI, 2.58–3.33) had a significantly increased risk, and if the pregnant patient fell into the range of severe obesity, the risk increased even more (BMI≥35 kg/m^2^; aOR, 4.14; 95% CI, 3.61–4.75) [32].

By conducting more studies and with a higher level of evidence, the different etiological factors of preeclampsia are understood, whose list increases, which in turn marks a common denominator of “endothelial cell dysfunction, intravascular inflammation and syncytiotrophoblast stress,” bringing with it the possible impact of maternal cardiovascular dysfunction, inadequate placentation, and possible involvement of infectious components.

## 6 Clinical manifestations

The clinical manifestations of preeclampsia vary from asymptomatic pictures to fatal complications for both the fetus and the mother. In severe cases, there may be renal, neurological, hepatic, or vascular system involvement [33].

Next, we will detail the main signs and symptoms associated with this pathology divided by organs and systems [4]: Neurological: headache, visual disturbances, hyperreflexia, clonus, or seizures. Hepatic: pain in the epigastrium or in the right upper quadrant. Hematologic: petechiae or dark urine color. Cardiorespiratory: dyspnea, tachypnea, chest pain or confusion. Uterus-placental and fetal: transvaginal bleeding, decreased fetal movements, uterus with increased tone.

ACOG [5], in its most recent practice bulletin, emphasizes that relying on maternal signs or symptoms for the diagnosis of preeclampsia is conflicting. Epigastric pain or severe pain in the right upper quadrant should not be attributed to alternative diagnoses. Likewise, headache should not respond to treatment with acetaminophen or be secondary to other etiologies [6]. On the other hand, the ISSHP [10] indicates that, in the presence of hypertension, the appearance of headache should be considered part of preeclampsia until proven otherwise.

A prospective and multicenter international study evaluated different variables to predict adverse pregnancy outcomes in 2023 patients with preeclampsia. Among their results, 52% of the patients reported at least one symptom, and of these, 5.2% had maternal or perinatal adverse effects compared to 5.3% of those who were asymptomatic. The authors found no correlation between clinical symptoms and unfavorable outcomes in pregnant women with preeclampsia [34].

In the case of HELLP syndrome in approximately 90% of cases, the main symptoms were pain in the right upper quadrant and general malaise, while in 50% of cases, they were nausea and vomiting. In the case of eclampsia, the main symptoms that precede seizures are neurological (severe, frontal, or persistent headache), blurred vision, photophobia or altered mental status [5].

## 7 Prediction of preeclampsia

The justification for predicting a pathology is to use more effective prevention strategies. There is no test or combinations of tests in the first or second trimester of pregnancy that can predict all cases of preeclampsia far from term or at term [5,7,8]. Two strategies have been studied, analyzed, and suggested to predict preeclampsia. One is based on clinical risk factors obtained with the questionnaire and the other on a screening with multiple factors (algorithm): clinical findings, mean arterial pressure, uterine artery pulsatility index determined by Doppler and blood serum placental growth factor (FCP).

Regarding the strategy based on risk factors, they are divided into high- and moderate-risk [6]. High risk: diabetes, chronic arterial hypertension, kidney disease, autoimmune diseases, abnormal uterine artery Doppler (positive), previous history of preeclampsia, or history of fetal or neonatal death associated with preeclampsia. Moderate risk: first pregnancy, family history of preeclampsia, multiple pregnancy, age greater than 40 years, Table 1. Unfortunately, only approximately 10% of women who develop preeclampsia far from term have clinical risk factors [10]. If we use the risk factors as recommended by ACOG [5] and that are similar to those of the World Health Organization (WHO) [6], it was shown in a study [35] that this detects 94% of preeclampsia in pregnancies at less than 32 weeks of gestation, 90% of preeclamptic women have less than 37 weeks of gestation and 89% of preeclamptic women have ≥37 weeks of gestation, but with 64% of false-positives. That is, a high percentage of preeclampsia is detected, but 6 out of 10 of them with these factors do not have preeclampsia.

**Table 1:** Clinical risk factors.

Maternal age> 35 years	RR = 1.5 (1.2–3.0)
Nulliparity	RR = 2.71 (1.96–3.74)
Previous history of PE	14.7% = 1 and 31.9% = 2
**Pregnancy interval**	**Ideal 1 to 5 years.**
Assisted reproduction	Double the risk
**Family History of PE**	**3**–**4 times more in sisters and daughters**
Obesity	BMI> 30 kg/m^2^ = 2–4 times more risk
Race	More risk: Afro-Caribbean and South Asian
**Comorbidities**	Higher risk: **Pregestational diabetes, chronic hypertension, kidney disease, SLE, APS**

The strategy based on the algorithm or with multiple factors should be performed between 11 and 14 weeks of gestation [1,35].

Prospective screening data with a prevalence of 2.9% of preeclampsia show that using this algorithm, 75% of preeclampsia is detected far from term and 43–47% of preeclampsia at term with a false-positive of 10% [36,37].

Adding to this algorithm the pregnancy-associated plasma protein A (PAPP-A) does not improve the results, so it is not recommended to add it [1]. The International Federation of Gynecology and Obstetrics (FIGO) recommends screening the first trimester (11–14 weeks) [1] to predict preeclampsia based on the algorithm (multiple factors) mentioned above. The disadvantages of screening based on the algorithm of several factors are as follows: a computerized program is required to calculate the risk and is not possible in many countries of the world, and especially is not at hand for the doctor in the office, it requires economic expenses to make the biochemical markers, requires experts to perform uterine artery Doppler and ultrasound equipment of adequate quality. In addition, it is necessary to do this between 11 and 14 weeks because after this period, its effectiveness is unknown. At that gestational age, many of the patients in low- and middle-income countries have not started prenatal care.

Organizations such as ACOG [7] and WHO [6] recommend screening for the risk of preeclampsia based only on the presence of risk factors obtained from the clinical history.

### 7.1 Preeclampsia prevention

Many drugs and strategies have been studied to reduce the possibility of hypertension during pregnancy. Diets have been studied, including low salt, vitamin, mineral and food intake, exercise, bed rest, use of calcium, aspirin, and low molecular weight heparin, among others, and some are still being investigated, such as metformin. No strategy or drug can prevent hypertension during pregnancy with high probability; however, there are approaches that have shown benefit.

We present strategies that can help under some conditions.

Calcium: Supplementation of calcium between 1 and 2.5 g per day after 20 weeks of pregnancy to women with a low or high risk of preeclampsia whose calcium intake is less than 900-600 mg/day (unlikely in many countries of the world, possibly in some populations or areas) is associated with a decrease in preeclampsia [8,38]. There is not enough evidence to suggest the ideal gestational age to start the use of calcium when it is justified [39]. In addition, using calcium before pregnancy or in the first half of pregnancy in patients with a history of hypertension during previous pregnancy shows no benefits [40].

When there is an indication to use calcium to prevent preeclampsia and there is also another beneficial strategy, such as aspirin, it can be used together.

### 7.2 Exercises

The adverse effects of exercise in pregnancy are unknown, except for the patient presenting with some disease that contraindicates it.

A systematic review of controlled trials shows that exercising reduces gestational hypertension (n = 5316; OR 0.61, 95% CI 0.43 to 0.85) and preeclampsia (n = 3322; OR 0.59, 95% CI 0.37 to 0.9). compared to not exercising [41]. To achieve these benefits, risk reduction, pregnant women should perform this activity for at least 140 min per week, including brisk walking, water aerobics, stationary cycling, or resistance training [41].

### 7.3 Aspirin

The concept of the imbalance between prostacyclin and thromboxane A_2_ as part of the pathogenesis of preeclampsia led researchers to use low-dose aspirin to prevent preeclampsia [42]. Multiple randomized trials and systematic reviews have been conducted to answer the question of the benefit of aspirin in preventing preeclampsia. A recent systematic review/meta-analysis [43] evaluated 45 randomized trials including 20,909 pregnant women, using aspirin with doses of 50–150 mg per day and using aspirin as a cutoff point before or after 16 weeks. They found benefits in the prevention of preeclampsia (RR, 0.57; 95% CI 0.43–0.75; P < 0.001; R^2^, 44%; P = 0.036) and severe preeclampsia (RR, 0.47; 95% CI, 0.26–0.83; P = 0.009; R^2^, 100%; P = 0.008) and for fetal growth restriction (RR, 0.56; 95% CI, 0.44–0.70; P < 0.001; R^2^, 100%; P = 0.044).; all when aspirin intake was started before 16 weeks. The preventive effect, according to this meta-analysis [43], was modest if it started after 16 weeks and had no effect on growth restriction. However, a meta-analysis of individual patients who involved 31 randomized studies and 32,217 pregnant women (published the same year as the previous one) found that the effect of prevention of preeclampsia and complications using aspirin is not affected if it starts before or after 16 weeks [44]. Many patients in the world and more in countries with fewer resources initiate prenatal control after the first trimester, so since there are benefits of aspirin initiated after 16 weeks, they would not be excluded.

The other question that arises is the dose to be used. A long multicenter, double-blind, placebo-controlled study [45] in women at risk using multiple screening (algorithm) [36,37] showed that taking aspirin at a dose of 150 mg per day at bedtime decreased the incidence of preterm preeclampsia (OR, 0.38; 95% CI, 0.20–0.74; P = 0.004). However, there were no differences between neonatal complications, nor did it decrease preeclampsia at term (>37 weeks) or preeclampsia in patients with chronic hypertension. For all the above, organizations such as ACOG [7], WHO [6] and US preventive services Task force [46] (based on the best evidence) suggest the use of aspirin at low doses (75–81 mg/day), starting as soon as the patient is taken in or it can be initiated at up to 28 weeks of pregnancy and the clinical risk factors already described in the prediction section can be used as criteria to offer it, and that dose is maintained until preeclampsia appears or until term. FIGO [1] and ISSHP [10] recommend 150 mg/day of aspirin, based on the findings of the ASPRE study [45]; that is, they recommend using these doses after performing the multiple screening (algorithm) between 11 and 14 weeks and if it is positive (at risk) to start prevention with that dose of aspirin. More studies using 150 mg of aspirin are needed, also studies comparing 150 mg versus 81 mg per day. On the other hand, there are findings of more obstetric bleeding using 150 mg/Day [4,6,47].

### 7.4 Low molecular weight heparin

Low molecular weight heparins have been associated with the prevention of preeclampsia; however, the results of the studies are very conflicting [48,49]. The most recent systematic review and meta-analysis [49] analyzed 15 randomized studies (2795 patients) and found a reduction in the development of preeclampsia (OR, 0.62; 95% CI, 0.43–0.90; P = 0.01), small for gestational age (OR, 0.61; 95% CI, 0.44–0.85; P = 0.003), and perinatal death (OR, 0.49; 95% CI, 0.25–0.94; P = 0.03), mainly if it was started before 16 weeks. Unfortunately, the quality of this evidence varies greatly and ranges from very low to moderate due to lack of randomization, imprecision, and great heterogeneity of the studies. Therefore, it is necessary to conduct randomized studies of adequate quality and quantity of patients before suggesting the use of low molecular weight heparins to prevent preeclampsia.

### 7.5 Management of preeclampsia

The international guidelines for the management of hypertensive disorders of pregnancy and preeclampsia are similar in many aspects, but there are important differences in terms of the definition of the severity of preeclampsia [5,50,51]. In addition, two major elements that aid in the definition and management of biomarkers are being introduced [52] and on the other hand, maternal morbidity prediction models (fullPIERS) [53], this affects the decisions of use of corticosteroids, antihypertensives, magnesium sulfate time and time of pregnancy termination. It should be noted that preeclampsia is progressive and tends to become more severe over time [4].

In the management of preeclampsia, we must consider the following: Possibility of predicting the second and third trimesters; it is preeclampsia; severity data present; fullPIERS or PREP, PREP-L, sFlt-1/PlGF with> 20% risk of maternal morbidity; what type of health facility are you in?; what is the gestational age and when should the delivery be?; medication to be used.

Prediction in the second and third trimesters: It was already presented and discussed in the previous session.

Definition of preeclampsia: As seen above, this must be clear, and a good definition is necessary. ISSHP [8] and FIGO [1,51] do not consider pulmonary involvement, and ACOG [5] does not consider utero-placental involvement in the definition. In addition, in the definition of preeclampsia, there are groups that include sFlt-1/PlGF> 38 pg/mL as a criterion [52]. All this has already been the subject of a comparison, and it has been determined that the inclusion of fetal compromise and angiogenic and antiangiogenic factors can be beneficial for the mother and fetus.

**Fig. 1:**
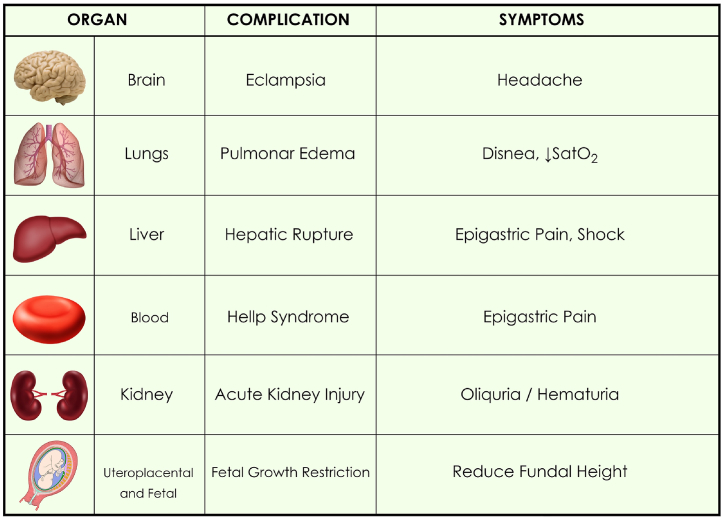
Symptoms and complications of severe preeclampsia.

Severity data: These include symptoms of involvement of each organ, such as headache, seizures, dyspnea, epigastric pain, shock due to subcapsular hematoma rupture, hematuria, oliguria, poor increase in uterine height, eclampsia, pulmonary edema, HELLP syndrome type 1, acute renal injury, rupture of subhepatic hematoma, IUGR, fetal death and laboratory parameters that require stabilization and immediate termination of pregnancy [4], Fig. 1.

Prediction of maternal and perinatal morbidity: Using fullPIERS, PREP, PREP-L, sFlt-1/PlGF calculators [52,53]. Early-onset preeclampsia is associated with severe maternal and perinatal complications. The fullPIERS (Preeclampsia Integrated Estimate of Risk) model has shown internal and external validity to predict maternal complications within 48 h for women admitted with preeclampsia at any gestational age. This ability to recognize women at higher risk of complications could help their management.

Health system and level of care: management is preferable where there is an intensive care unit or special care unit, availability of other specialties, complete laboratory and imaging, blood bank. If these services are not available, after stabilizing the patient, the patient should be referred. If there are criteria of severity, with the initial management of antihypertensives, magnesium sulfate, fetal maturation, double venous cannulation, and bladder catheter [51].

Ideal gestational age for delivery. Numerous investigations and systematic reviews have been published to define the best moment:

**<24 weeks** [54]. In an investigation with 55 women with severe preeclampsia younger than 24 6/7 weeks with expectant management, 52 died intrauterus, 1 perinatal death and 2 lived with some psychomotor or motor deficits and mothers with HELLP syndrome, eclampsia, transfusions.

**24**–**34 weeks**: In the MEXPRE study [55], 267 pregnant women with severe preeclampsia were studied: 133 were randomized to immediate post-corticosteroid interruption, and 134 were randomized to expectant management. Perinatal mortality was similar, and maternal mortality was similar, but in the expectant group, there was more premature detachment of the placenta and small for gestational age. In the TOTEM [56] study, 56 pregnant women were evaluated with expectant management and delivery after fetal maturation with corticosteroids, showing no neonatal benefits.

**34**–**37 weeks**: The HYPITAT 2 study [57], in this study with 703 pregnant women with preeclampsia without data of severity between 34 and 37 weeks, did not significantly decrease maternal morbidity but tripled the risk of fetal distress. The subsequent study of neonates at 2 and 5 years showed no differences in development. The PHOENIX [58] study studied 901 women with mild preeclampsia, demonstrating that maternal morbidity decreased in active management and increased neonatal morbidity.

**> 37 weeks**: HYPITAT 1 [59]. In this study, 756 women with preeclampsia without data of severity of more than 36 weeks for active or expectant management were studied, and it was demonstrated that active management reduces the risk of maternal morbidity.

Medication to be used: Antihypertensives: can be used to maintain a stable pressure and avoid the hypertensive crisis of systolic blood pressure >160 mm Hg, diastolic blood pressure> 110 mm Hg, or both: Oral nifedipine: 10 mg every 20 min for a maximum of 50 mg. Intravenous Labetalol: 20 mg initially and if necessary to repeat in 15–20 min, it is doubled to 40 mg, and if 15–20 min later, it is maintained in hypertensive crisis, it is now doubled to 80 mg, and this last dose can be repeated twice more every 15–20 min if necessary. Intravenous hydralazine should be used 5–10 mg intravenously every 20 min for 3 to 5 doses if necessary. Magnesium sulfate: for prevention of eclampsia and fetal neuroprotection in children younger than 32 weeks. In addition, adding that with impregnation and receiving at least 8 h with magnesium sulfate prior to delivery, there would be no benefits of continuing with magnesium sulfate postpartum. The protection is similar if it is removed [60], if it is left for 6 h [61], for 12 h or if it is left for 24 h postpartum [62].

Corticosteroids: for fetal lung maturation preferably with betamethasone 12 mg intramuscularly and repeated in 24 h or dexamethasone 12 mg intramuscularly and repeated in 24 h [63].

### 7.6 Maternal and perinatal complications of preeclampsia

The main complications occur in 6 target organs, including pulmonary edema and utero-placental involvement [51], Fig. 1. We will mention the 6 most important:

Eclampsia is defined as the appearance of one or more generalized tonic-clonic seizures not related to other medical conditions in women with hypertensive disorder of pregnancy. There is loss of autoregulation of cerebral blood flow, blood–brain barrier damage, edema and if it is associated with HELLP syndrome, possible cerebral hemorrhage that is the main cause of death [64] and that can be at the subarachnoid, intraparenchymal or intraventricular level. In management, several schemes of magnesium sulfate are presented, and it is the best anticonvulsant for these cases.

**Fig. 2:**
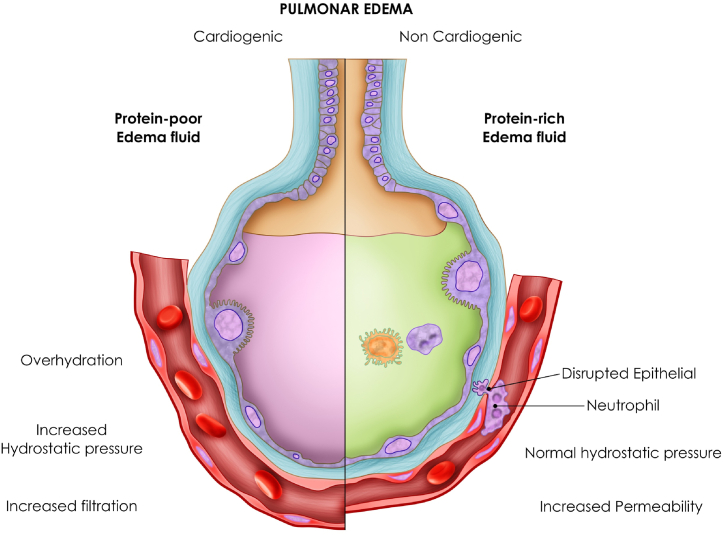
Pulmonary edema is the final accumulation of fluid in the pulmonary alveoli. There are two types: cardiogenic (also called hydrostatic) the main in preeclampsia and noncardiogenic (due to increased permeability).

Pulmonary edema is the final accumulation of fluid in the pulmonary alveoli. There are two types: cardiogenic (also called hydrostatic) 64.3% in preeclampsia and noncardiogenic (due to increased permeability) 14.3% in preeclampsia [65]. It is essential to distinguish them for management: in cardiogenic cases, diuretics are used to reduce afterload, and in noncardiogenic cases, they require mechanical ventilation with low tidal volume. It arises as a result of 3 conditions, increased pressure, overhydration and endothelial damage, Fig. 2.

Hepatic rupture is the spontaneous rupture or distention of the Glisson capsule in women with severe preeclampsia and HELLP syndrome and is due to the distension and tension produced by a hematoma or hepatic subcapsular edema. This rupture is caused by periportal hemorrhage and intravascular fibrin deposition in the hepatic sinusoid, obstruction and massive intravascular congestion that contributes to increased hepatic pressure and necrosis that leads to subcapsular and intraparenchymal hemorrhage. After endothelial damage, there is extravasation of red blood cells, thrombosis, hemorrhage, hematoma formation and rupture [66]. The right lobe is involved in 75% of cases and the accuracy of the degree of liver damage should be determined with the AAST liver trauma classification (there are 6° from grade I: subcapsular hematoma that occupies less than 10% of the surface of the liver. lobe up to grade VI with hepatic avulsion) or World Society of Emergency Surgery (WSES) grade I-IV and its multiple management based on liver packaging until liver transplantation [67].

### 7.7 HELLP syndrome

**Fig. 3:**
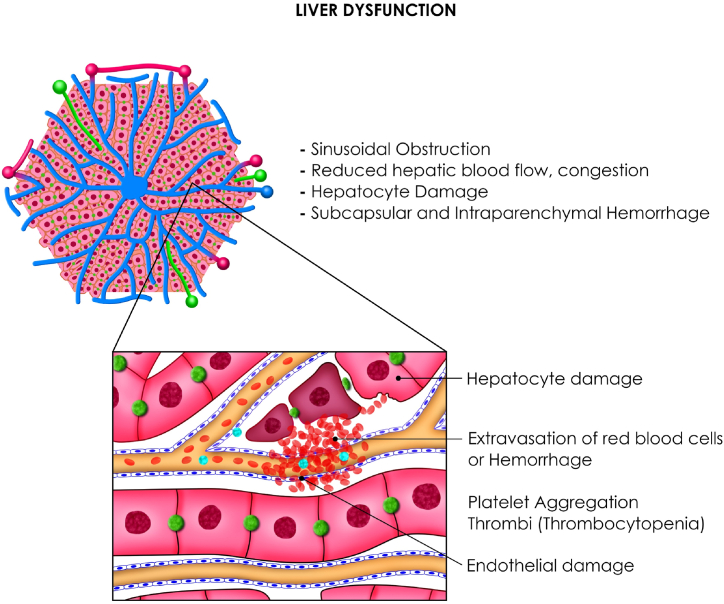
HELLP syndrome is one of the most severe complications of preeclampsia, Hepatic dysfunction with platelet consumption is the most characteristic in HELLP syndrome.

HELLP syndrome is one of the most severe complications of preeclampsia, causing great maternal and perinatal morbidity and mortality. After having defined that a patient has preeclampsia, she must have the triad: (H) Hemolysis, elevated liver enzymes (EL) and thrombocytopenia (TC). In management, the use of dexamethasone has been shown to improve the number of platelets and decrease hospital time [68]. Fig. 3.

### 7.8 Acute kidney injury (AKI)

The International Organization for Kidney Disease: Improving Global Outcomes (KDIGO) defines AKI in 2020 as a sudden decrease in the glomerular filtration rate (GFR) manifested by an increase in serum creatinine or oliguria between 48 h and 7 days.), with the severity (stage) of AKI determined by the severity of the increase in creatine or oliguria [69]. The anatomopathological findings include glomeruloendotheliosis, podocyturia and microangiopathic thrombosis [69], in addition to the presence of hematuria as a sign of severity. Classification in AKI stages 1, 2 and 3 of the last classification and its management may require replacement therapy and hemodialysis [69].

### 7.9 Uterine-placental involvement

The definition of IUGR is based on the calculation of the weighted fetal ultrasound and the Doppler velocimetry of the uterine, umbilical, and middle cerebral arteries [70]. To see the degree of involvement (I-IV), ductus venosus [71] was added. IUGR is caused by uteroplacental insufficiency that can increase perinatal morbidity and mortality. Preeclampsia of early onset, that is, before 34 weeks [71], is mostly associated with IUGR. Preeclampsia and IUGR are associated with small placentas in childbirth and show combinations of villus hypermaturity, infarcts, and decidual vasculopathy.

## 8 Conclusions

Preeclampsia is a multisystem disorder originating at the level of the uterus and placenta, and we know much about the damage it produces at the maternal-fetus-neonatal level. We do not know how to predict it accurately, and therefore, we do not know biochemical or biophysical markers that allow us to act before clinical manifestations or damage to the mother and her child. Despite decades of researching how to prevent this disease, we are far from having a nonpharmacological or pharmacological measure that allows us to avoid a high percentage of this pathology. We use acetylsalicylic acid at low doses to prevent a low percentage, especially in patients with far from term preeclampsia that, as we know, are the ones that present the least, although they are the ones that present more complications. Management consists basically of treating hypertension, avoiding seizures, interrupting pregnancy, and treating complications. Maternal complications of preeclampsia are not limited to pregnancy; today, we know that they have more possibilities of cardiovascular and metabolic disorders for the rest of their lives. Evidence shows that women with preeclampsia are twice as likely to develop chronic hypertension, type 2 diabetes mellitus and hypercholesterolemia compared to those without hypertension. It is necessary to continue research on preeclampsia to learn more about this pathology, to have better maternal and perinatal outcomes and to make the difference between preterm and term preeclampsia.

It is necessary to continue doing research focused on the use of biomarkers (fetus, placenta, and mother). It is necessary to improve the prediction and prevention of preeclampsia, in addition, better research is needed in the long-term postpartum follow-up. Currently, the safest approach is the termination of pregnancy.

## Author contribution statement

Paulino E. Vigil-De Gracia, Jorge Collantes-Cubas: Conceived and designed the experiments; Performed the experiments; Analyzed and interpreted the data; Contributed reagents, materials, analysis tools or data; Wrote the paper.

Carlos Vargas, Joanne Sánchez: Conceived and designed the experiments; Performed the experiments; Contributed reagents, materials, analysis tools or data; Wrote the paper.

## Funding statement

This research did not receive any specific grant from funding agencies in the public, commercial, or not-for-profit sectors.

## Data availability statement

No data was used for the research described in the article.

## Declaration of interest’s statement

The authors declare no competing interests.
